# Responses of Ileal and Fecal Microbiota to Withdrawal of Pancreatic Enzyme Replacement Therapy in a Porcine Model of Exocrine Pancreatic Insufficiency

**DOI:** 10.3390/ijms231911700

**Published:** 2022-10-02

**Authors:** Julia Hankel, Anne Mößeler, Clara Berenike Hartung, Silke Rath, Lisa Schulten, Christian Visscher, Josef Kamphues, Marius Vital

**Affiliations:** 1 Institute for Animal Nutrition, University of Veterinary Medicine Hannover, 30173 Hanover, Germany; 2 Institute for Animal Nutrition and Dietetics, Vetsuisse-Faculty, 8057 Zürich, Switzerland; 3 Microbial Interactions and Processes Group, Helmholtz Centre for Infection Research, 30628 Braunschweig, Germany; 4 Institute for Medical Microbiology and Hospital Epidemiology, Hannover Medical School, 30625 Hannover, Germany

**Keywords:** minipigs, exocrine pancreas insufficiency, maldigestion, gut microbiota, small intestine

## Abstract

Little is known regarding the interplay between microbiota and pancreas functions in humans as investigations are usually limited to distal sites, namely the analyses of fecal samples. The aim of this study was to investigate both ileal and fecal microbiota in response to pancreatic enzyme replacement therapy (PERT) in a porcine model of exocrine pancreatic insufficiency (EPI). PERT was stopped for ten days in ileo-cecal fistulated minipigs with experimentally induced EPI (*n* = 8) and ileal digesta as well as fecal samples were obtained before withdrawal, during withdrawal and after the reintroduction of PERT. Profound community changes occurred three days after enzyme omission and were maintained throughout the withdrawal phase. A reduction in α-diversity together with relative abundance changes in several taxa, in particular increases in *Bifidobacteria* (at both sites) and *Lactobacilli* (only feces) were observed. Overall, dysbiosis events from the ileum had accumulating effects in distal parts of the gastrointestinal tract with additional alterations occurring only in the colon. Changes were reversible after continuing PERT, and one week later, bacterial communities resembled those at baseline. Our study demonstrates the rapid and profound impacts of enzyme withdrawal in bacterial communities, contributing to our understanding of the interplay between pancreas function and microbiota.

## 1. Introduction

Diseases of the pancreatic parenchyma, including chronic pancreatitis, cystic fibrosis, and a history of extensive necrotizing acute pancreatitis, can result in exocrine pancreatic insufficiency (EPI), which is defined as a decline in active exocrine pancreatic enzymes resulting in the maldigestion and malabsorption of nutrients [1]. Impaired exocrine pancreatic function is associated with changes in colonic microbiota composition and diversity in humans [2] and alters microbial-derived metabolites [3]. Even in healthy individuals, exocrine pancreatic function, measured by fecal pancreatic elastase levels, is associated with fecal microbiota composition [4]. On the other hand, gut microbiota in patients with chronic pancreatitis seems to be only moderately affected by pancreatic exocrine function [5]. Increasing experimental evidence suggests a link between microbiota dysbiosis and various pancreatic diseases; however, the exact roles of microbiota in etiology are yet to be elucidated [6]. Potential mechanisms involve immune system modulations and the production of bacterial metabolites, such as short-chain fatty acids [7].

Investigations into microbiota under impaired exocrine pancreatic function in humans are limited to analyses of stool samples. However, EPI is often associated with small intestinal bacterial overgrowth (SIBO) [8], a condition that describes a state of microbial dysbiosis in the small bowel, characterized by an immigration and growth of colonic bacteria [2,9]. SIBO complicates pancreatic diseases and its treatment leads to an amelioration of symptoms [8]. It is believed that SIBO is triggered by an altered availability of substrates for microbial growth; however, the exact mechanisms causing these conditions are not fully understood [2]. The direct treatment of SIBO includes the oral administration of antibiotics, resulting in improved fat digestion and absorption [10]. Usually, pancreatic enzyme replacement therapy (PERT) is the treatment of choice for EPI as it adjusts for malnutrition improving gastrointestinal symptoms and quality of life [11]. However, not all patients respond to PERT, and the reported symptoms under SIBO are often refractory to PERT [8]. SIBO might play a pivotal role in the persistence of steatorrhea in individuals adequately supplemented with enzymes, accompanied by a malabsorption of bile acids and an alteration of intestinal permeability during EPI [12]. Therefore, a better understanding of gut microbiota under EPI can potentially reveal biomarkers for monitoring treatment efficacy and assist in the development of new therapeutic strategies [12]. In this context, microbiota at proximal sites, such as the ileum, should be investigated since these bacteria, due to their proximity to the organ, are expected to be more affected by pancreas malfunction compared with colonic microbiota.

Based on the high similarity of the digestive processes with humans and the feasibility to ligate the pancreatic duct, pigs, especially minipigs, are often used as EPI models [13,14,15]. The pancreatic duct ligated minipig with ileo-cecal re-entrant fistula enables us to study the effects of this disease on digestion with the aim of optimizing treatment and dietetic measures in humans [16,17]. The animal model of the pancreatic duct ligated minipig with ileo-cecal re-entrant fistula used in the present study gave us the opportunity to repeatedly collect ileal digesta in addition to fecal samples. With this model to hand, we investigated the temporal dynamics of ileal and fecal microbiota under EPI and their response to PERT.

## 2. Results

**Figure 1:**
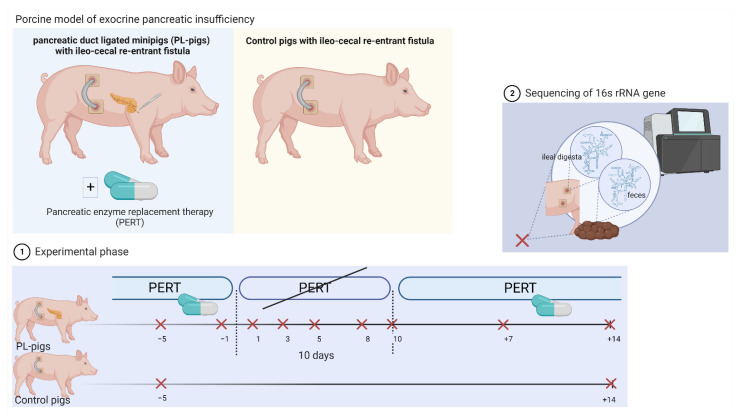
Schematic representation of the study set-up that used a porcine model of exocrine pancreatic insufficiency. During the experiment, pancreatic enzyme replacement therapy (PERT) was stopped and again re-administered in pancreatic duct ligated minipigs (PL-pigs). Ileal and fecal samples were taken during PERT (*n* = 2 timepoints; days −5 and −1), during withdrawal of PERT (*n* = 5 timepoints; days 1, 3, 5, 8, 10) and again after re-administration of PERT (*n* = 2 timepoints; days +7, +14). Samples of ileal digesta and feces of ileo-cecal fistulated healthy minipigs (Control pigs) were taken at the beginning and end of the experiment. The sequencing of the 16S rRNA gene was conducted in obtained samples for microbiota analyses. The figure was created using biorender.com (accessed on 26 September 2022).

**Figure 2:**
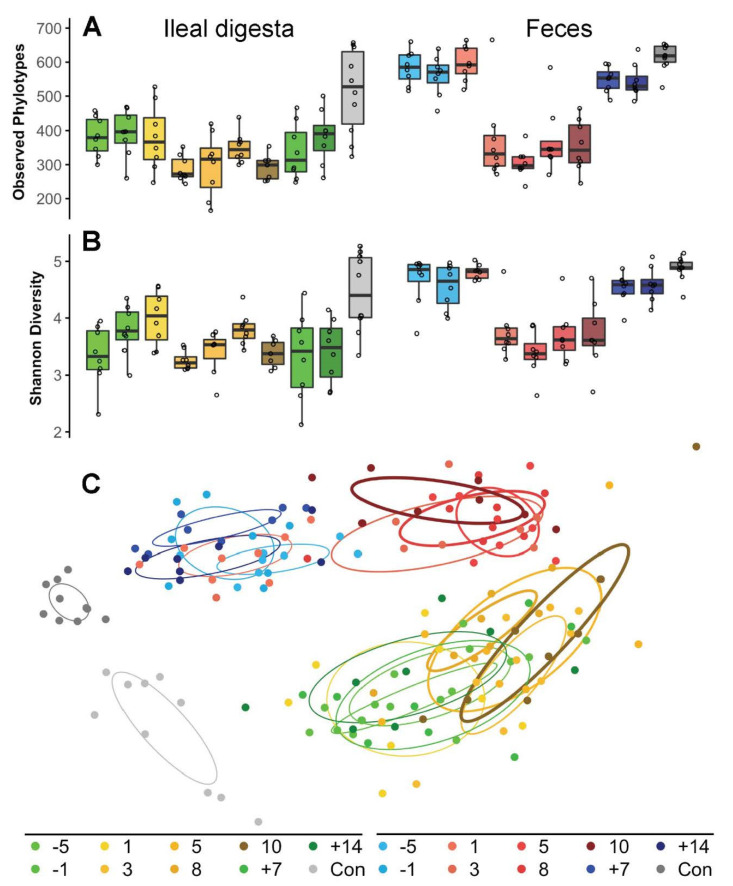
Diversity alterations in bacterial richness (number of phylotypes) (**A**) and Shannon diversity (**B**) as well as ordination analysis of community composition (non-metric multidimensional scaling analysis) (**C**) in ileal digesta and fecal samples at baseline, during enzyme withdrawal and after enzyme reintroduction. Key to colors indicating sampling site (ileal digesta: shades of yellow/green and feces: shades of blue/red) and sampling time points (days before (−)/during/after(+) PERT withdrawal) is provided at the bottom. Results from Control pigs (Con) are also provided (grey colors).

An outline of our experimental setup is shown in Figure 1, and the basic characteristics of animals used in this study are given in Table 1. At baseline, α-diversities, defined as bacterial richness and the Shannon index, in fecal samples of pancreatic duct ligated minipigs (PL-pigs) under pancreatic enzyme replacement therapy (PERT), were similar to those of fecal samples derived from healthy control minipigs (Control pigs) (Figure 2A,B). α-diversity values for ileal digesta showed high variations, especially in control animals, and tended to increase in the latter; however, differences were not significant between PL-pigs and Control pigs. Diversities in ileal digesta were lower compared with feces in PL-pigs. The discontinuation of enzyme therapy caused a significant reduction in α-diversities in the ileal digesta and feces of animals compared with baseline, three days after withdrawal of PERT. Changes were more pronounced in fecal samples and Shannon diversity in ileal digesta hardly changed (Figure 2A,B). A strong response was also reflected in community compositions that rapidly changed (3 days) at both sites after enzyme withdrawal and maintained their structures throughout this phase until day 10 (Figure 2C). A stronger shift was observed in fecal samples than those derived from ileal digesta. One week after reintroduction of PERT, microbiota re-established their structures, which were comparable to those at baseline, as did α-diversities (Figure 2). Resilience based on diversity was less in the ileum and it took 14 days of PERT to detect a similar richness as compared with baseline, whereas Shannon diversity stayed low until to the end of experiment. Overall, community structures differed distinctly between the two sites throughout the experiment in PL-pigs. Furthermore, ileal and fecal bacterial community structures from animals under PERT differed from those of healthy animals.

**Figure 3:**
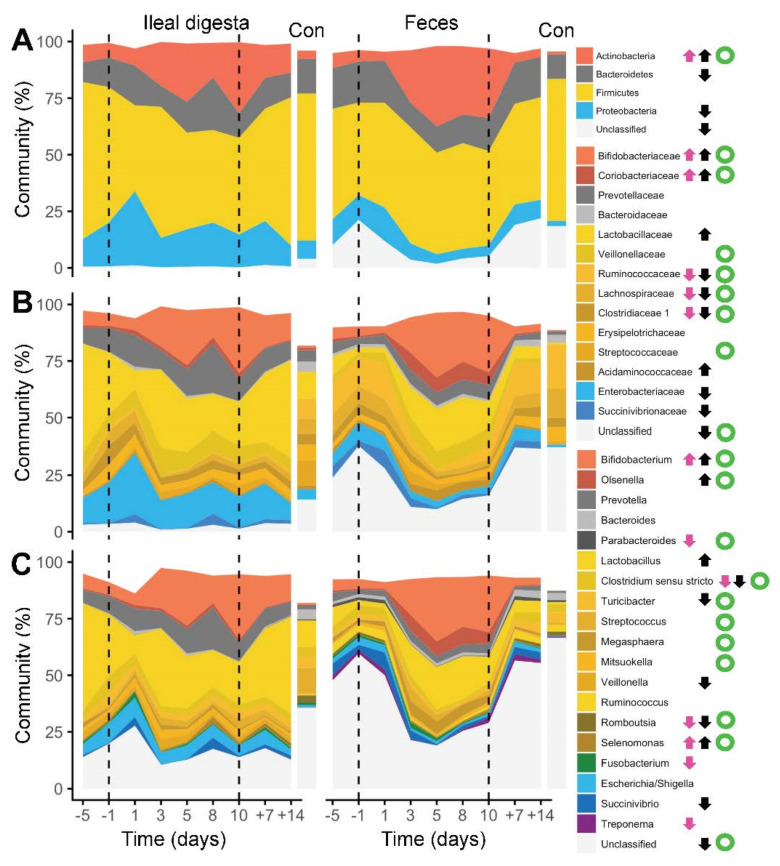
Alterations of individual bacterial phyla (**A**), families (**B**) and genera (**C**) in ileal digesta and fecal samples during the experiment (dashed lines highlight period of enzyme withdrawal). Data from Control pigs (Con) is also provided. Arrows indicate significant changes in relative abundance of taxa before and after PERT depletion (pink: ileal digesta, black: feces), whereas green open circles show significant correlations (FDR corrected Spearman’s rho < 0.05) between the two sites.

Subsequent analyses revealed that many taxa altered their abundance upon enzyme withdrawal. At the phylum level, *Actinobacteria* increased at both sites, which was mainly triggered by *Bifidobacteria* (Figure 3). Additionally, an increase in *Lactobacillaceae* was seen in fecal samples, whereas other major *Firmicutes* families decreased. A decrease in the abundances of the *Bacteroidetes* and *Enterobacteriaceae,* as well as *Succinivibionaceae* (the latter two families belonging to *Proteobacteria)*, were detected in feces only, and overall, more significant changes were detected in fecal samples compared with ileal digesta. In general, the abundance of many major taxa correlated between the two sites indicating that their response was similar in the ileum and the colon.

Detailed investigations revealed 18 phylotypes that changed their abundances in a similar manner at both sites, whereas 3 and 14 phylotypes showed significant changes only in the ileal digesta and colon, respectively (Table 2). Significant correlations in abundances between the two sites were observed for 23 phylotypes. Most evident was the increase in several *Bifidobacterium*-associated phylotypes, where one phylotype annotated as *B. thermophilum* showed high abundances at both sites. Furthermore, abundances of several phylotypes assigned to *Lactobacillus* increased in fecal samples during enzyme withdrawal, and abundances of phylotypes assigned to *Prevotellaceae* increased at both sites.

## 3. Discussion

Data on the correlation between microbiota and pancreatic diseases are still scarce, and conclusions were mainly drawn based on the analysis of the stool samples [18]. Since pancreatic juice shows antibacterial activity [19] and the excretion of pancreatic enzymes occur in the duodenum, alterations of microbiota composition are already expected to occur in the small intestines. EPI is often associated with SIBO, which can complicate the disease, indicating that major pathological mechanisms, which potentially include microbiota, can happen in the small rather than in the large intestine [8]. While microbiota under PERT in a porcine model of EPI have already been investigated, these analyses were limited to fecal samples [13], leaving the small intestine largely unexplored. Furthermore, no dynamics in community alterations are available. Previous cultivation-based studies enumerating selective bacterial taxa (Gram-negative anaerobic bacteria, *Escherichia coli*, *Clostridium perfringens*, enterococcus/streptococcus and lactobacilli) in the same porcine model indicated that bacterial counts in the ileal chyme did not significantly differ between control pigs and pancreatic duct ligated minipigs, whereas fecal samples of pancreatic duct ligated minipigs showed greatly increased bacterial densities (factor 10 to 100 based on cfu and LPS measurements) [20,21,22]. However, higher contents of lipopolysaccharides in ileal chyme were detected in pancreatic duct ligated minipigs, indicating higher bacterial loads. To the best of our knowledge, this is the first study on EPI to investigate the microbiota of the small intestine in the same animals with and without PERT over time using cultivation-independent methods. Our unique experimental setup allowed us to monitor temporal dynamics of bacterial community compositions during PERT withdrawal in both small intestine and fecal samples.

Community composition at both sites rapidly changed after enzyme withdrawal and showed resilience after the reintroduction of enzyme therapy, where final microbiota resembled those at baseline. Additionally, other disturbances, such as pronounced dietary alterations or antibiotic treatment can perturb the composition of microbiota within a few days [23]. For instance, it was repeatedly reported that changes in host diet induced relative abundance changes in gut microbiota members within one or two days [24,25,26]. Longitudinal studies of the adult intestinal microbiota have shown rapid responses to perturbations such as antibiotic treatment and dietary changes [27]. However, gut microbiota usually show a high resilience, reverting to their original structure during dietary interventions [25,28]. This has also been observed for antibiotic treatment, where gut microbiota changed within 3–4 days after drug initiation and communities began to return to their initial state one week after the end of treatment [29]. Our results on the temporal dynamics of microbiota under PERT withdrawal and the reintroduction of PERT in EPI can therefore be considered as an acute disturbance, similar to other major disturbance events that result in profound alterations of microbial communities, maintaining a high potential for resilience. Microbiota changes during longer-term “press” disturbances, such as untreated and chronic insufficiencies, and how this impacts resilience, are currently unknown [30].

In this study, we demonstrated that the discontinuation of PERT caused a significant reduction in α-diversities that rapidly occurred on both sites. Similar reductions in diversity, as well as significantly influenced microbiota phylotypes, were observed in stools of children with cystic fibrosis compared with healthy controls [31]. Furthermore, fecal elastase levels were positively associated with markers of α-diversity demonstrating that exocrine pancreatic function is associated with gut microbiota diversity [3]. Under PERT, bacterial diversity in the feces of PL-pigs returned to near healthy levels as measured in control pigs, which was already observed by others using a similar porcine model of exocrine pancreatic insufficiency [13], as well as in humans [32]. In terms of individual taxa, we found a multitude changing their abundances upon PERT withdrawal, which is supported by previous studies in humans and animal models [3,4,13]. For instance, we observed an increase in *Prevotellaceae*-associated phylotypes at both sites during enzyme withdrawal (two additional for ileal digesta), while two phylotypes of the *Bacteroidales* (both sites) decreased, which is in line with a reported increase in *Prevotellaceae* accompanied by a decrease in *Bacteroidaceae* in humans under impaired exocrine pancreatic function compared with healthy controls [4]. While those taxa did not change their overall abundances in our study, the major associated phylotypes mentioned above all displayed the same pattern. Pigs show remarkable similarities with humans in terms of both gut anatomy and microbial composition and are therefore considered a superior model for microbiota research over rodents; however, several differences from human gut microbiota exist [33]. For instance, *Lactobacilli*, which responded well in our study, were shown to be overall more abundant in pigs than humans. Additionally, one of the most prominent markers of our study, namely *Bifidobacteria*, was not found to be altered during EPI in human stools [3,4]. This taxon increased during enzyme withdrawal at both sites with phylotypes closely matching *B. thermophilum* and *B. pseudolongum*, which are often isolated from feces of pigs [34], forming the most major part. Additionally, during PERT, the PL-pigs displayed a much higher proportion of *Bifidobacteria* compared with controls. In healthy adult humans, *Bifidobacteria* form about 3–6% of the total bacterial population in the colon [35] and relative abundances of 2.5% (ileum) and 3–4% (feces) were reported in pigs [36]. A similar increase in *Bifidobacteria* concentrations in the feces of minipigs in the presence of EPI without PERT was described by other researchers [13], and a study investigating EPI-affected dogs came to the same conclusion [37]. The latter reported an abundance in fecal *Bifidobacteria* of 19.5% and 31.6% in EPI with and without PERT, respectively, and much lower levels in healthy controls (2.8%). We hypothesize that the differences between human and animal models are due to the distinct substrate availabilities for bacteria, either due to differences in dietary compositions or differences in residual pancreatic function of diseased individuals. Fecal chymotrypsin values, which are used as indirect tests of pancreatic function, were in the range of 2.4–26.9 U/g in our healthy minipigs, while all pancreatic duct ligated minipigs had values of <0.5 U/g. Chymotrypsin levels in healthy children are 10 to 95 U/g (mean: 52.2 ± 3.7) and range from 0.2 to 3.0 U/g, with a mean value of 1.5 ± 0.3, in cystic fibrosis children suffering from pancreatic insufficiency [38]. In general, values between 3 and 6 U/g in stools for fecal chymotrypsin are usually considered to indicate EPI in humans [39]. Thus, the enzyme production of our pancreatic duct ligated minipigs is much lower than in humans being diagnosed with EPI, which probably leads to a greater impairment of the digestive capacity of the animal model profoundly changing the environment for bacterial growth.

Malnutrition, a hallmark of EPI, often comes along with an altered redox potential, and a depletion of obligate anaerobes with a concomitant increase in oxygen-tolerant bacteria, such as *Bifidobacteria* and *Lactobacilli*, in such circumstances as those previously described [40]. Under normal conditions, the reflux of chyme from the cecum into the ileum is prevented by the sphincter ilei muscle. The re-entrant fistula bypasses this mechanism and a reflux affecting the ileal microbiota is possible in theory. However, EPI and control pigs displayed extensive differences in their composition of the ileal microbiota (all pigs were fistulated), demonstrating the strong effects of EPI masking potential bias due to the fistula. As healthy fistulated Control pigs had a very low proportion of *Bifidobacteria,* an influx of oxygen into the intestinal lumen through the fistula can be excluded causing the increased abundances of these bacteria in PL-pigs. Other gastrointestinal tract diseases associated with malabsorption, such as inflammatory bowel disease or short bowel syndrome, have been associated with a higher prevalence of *Bifidobacteria* and *Lactobacilli* in several studies [41,42]. Under EPI, nutrients from diet are less available to the host and can be used by intestinal bacteria for growth. Thus, bacteria and the host compete for nutrients, and the higher abundances of specific bacterial taxa are likely a direct consequence of the increased substrate availability under EPI, promoting an environment in which *Bifidobacteria* (small and large intestine) and *Lactobacilli* (only large intestine) had a growth advantage. Another study from our institution observed an increase in the relative abundances of *Bifidobacteriaceae* and *Lactobacillaceae* in pigs whose diet was characterized by higher starch availability in the small and large intestines due to the varying grinding intensity of cereals used in their diets [43], supporting the hypothesis that increased starch availability in EPI promotes the growth of those taxa. While steatorrhea is a typical symptom in EPI, starch concentration in feces is very low, which may lead to the assumption that overall starch digestion is not affected in EPI patients. Ileal digestibility of starch is, however, markedly lower in untreated EPI [16] reaching values of around 70% [44]. The high amounts of residual starch are therefore available for microbes, which are supported by high fermentation rates observed in the small intestines in untreated patients, as indicated by in vitro studies [45], as well as by the development of SIBO indicated by breath testing of hydrogen or methane [46]. Furthermore, analyses of short-chain fatty acids (SCFAs) in ileal chyme obtained from animals of the same porcine model indicated that fermentation rates tend to be higher in PL minipigs compared with healthy controls accompanied by SCFAs composition differences between the two animal groups [20]. *Bifidobacteria* are primary degraders of various complex carbohydrates [47,48], and it was reported that indigestible carbohydrates, such as resistant starch, short-chain fructooligosaccharides, soy oligosaccharides and galactooligosaccharides, increased the proportion of this taxon in both humans and pigs [49,50]. Plant proteins, such as pea and soy protein, also have a bifidogenic effect [51,52]. The maintenance diet of our minipigs consisted of cornmeal, wheat flour, and soy extraction meal (4.4% disaccharides and 42.8% polysaccharides) likely provided ideal growth conditions for those bacteria, in particular in PL-pigs. *Bifidobacteria* are generally considered probiotic and health-promoting [53]. Our study indicates that very high abundances of this taxon in the ileum might be detrimental under specific conditions and the role of *Bifidobacteria* should therefore be assessed context dependent.

Importantly, an increase in *Bifidobacteria* was observed in the ileal digesta and feces, hinting that the over-proportional growth of this taxon during enzyme withdrawal already took place in the small intestine. In general, we observed a multitude of taxa being differentially abundant at both sites, and we speculate that their abundance alterations primarily occurred in the small intestine, and respective results in feces can reflect proximal events. This is supported by the fact that the abundances of only a few taxa were altered in ileal digesta. On the other hand, various taxa displayed differential abundances without PERT solely in fecal samples. Most prominent was the genus *Lactobacillus*, indicating that enzyme withdrawal opens a growth niche for those bacteria in the colon, while not affecting their growth in upper parts. Thus, fecal samples allow for the assessment of EPI and monitoring of treatment, as dysbiosis events happening at various sites in the gastrointestinal tract are reflected in this specimen. However, the results obtained from fecal samples do not point to this site, where dysbiosis initially occurred.

Our study provides new insights into the impact of EPI on gut microbiota at two intestinal sites. However, more function-centric investigations encompassing comprehensive multi-omics studies together with detailed data on organ performance have to be performed in order to obtain detailed insights into gut microbiota–pancreas crosstalk.

## 4. Materials and Methods

### 4.1. Animal Model

At the Institute for Animal Nutrition of the University of Veterinary Medicine Hannover, Foundation, a total of 13 adult female Göttingen minipigs (Ellegaard Göttingen Minipigs A/S, Dalmose, Denmark) of a porcine model of exocrine pancreatic insufficiency were used for the experiment [54]. All animals were fitted with an ileo-cecal re-entrant fistula, according to a modified method of Easter and Tanksley [55], and Hazem and Drochner [56] allowing ileal digesta to be sampled over time. The *ductus pancreaticus accessorius* was additionally ligated in 8 out of 13 animals in order to induce EPI. The pancreatic duct ligation and the insertion of the fistula were carried out in the same operation. For this purpose, a laparotomy was performed under general anesthesia in the linea alba. First, the pancreas was exposed, the *ductus pancreaticus accessorius* was ligated twice (towards the organ and towards the intestine) and cut between the ligatures. In all animals, the terminal ileum was transected orally to the *ostium ileale* and both opened sites of the intestine were closed with sutures. Titanium fistula tubes were then inserted into incisions made at the sutured end of the ileum and the tip of the cecum and pushed through the abdominal wall on the right side of the body from the inside to the outside. The re-entrant fistulae were then fixed caudally of the ribs with a counterpart and connected by use of a flexible silicon tube.

Pancreatic exocrine function was evaluated by determining fecal chymotrypsin activity after surgery (test kit purchased from Immundiagnostik AG, Bensheim, Germany). Pancreatic duct ligated minipigs with a chymotrypsin activity <0.900 U/g feces were defined as PL-pigs (Table 1). Repeated samples of ileal digesta and feces were taken during pancreatic enzyme replacement therapy (PERT, porcine multienzyme product at a dosage of 300,000 IU lipase, 187,500 IU amylase and 12,000 IU protease per meal) (*n* = 2 timepoints; days −5 and −1), during withdrawal of PERT (*n* = 5 timepoints; days 1, 3, 5, 8, 10) and again after re-administration of PERT (*n* = 2 timepoints; days +7, +14) (Figure 1). The remaining five ileo-cecal fistulated healthy minipigs served as controls. Samples of ileal digesta and feces of Control pigs were taken at the beginning and end of the experiment.

Animals had free access to water and were fed a commercial diet twice a day (Altromin 9021, Altromin Spezialfutter GmbH & Co. KG, Lage, Germany) consisting of cornmeal, wheat flour, and soy extraction meal (chemical composition: 11.3% moisture, 17.5% crude protein, 4.5% crude fat, 4.0% crude fiber, 6.2% crude ash, 3294 kcal ME/kg diet as well as 4.4% disaccharides and 42.8% polysaccharides). Green meal and wheat bran were removed from the original receipt.

### 4.2. Microbiota Analyses

DNA extraction and library preparation were carried out according to Rath et al. [57] where the V1V2 region of the 16S rRNA gene was amplified using a two-step approach. In detail, DNA was extracted using the FastDNA™ SPIN Kit for Soil (MP Biomedicals, Heidelberg, Germany) according to the manufacturer’s instructions and purified using the QIAquick PCR Purification Kit (Qiagen, Hilden, Germany). One microliter of template DNA from the extract was amplified using the PrimeSTAR HS DNA Polymerase (Takara, Otsu, Shigu, Japan), which served as the template for a second PCR step adding the two indices and Illumina adapters. For detailed PCR conditions see Rath et al. [53]. Amplicons of different samples were equimolarly pooled and sequenced on Illumina MiSeq (2 × 300 bp). Raw reads were processed according to Rath, Heidrich, Pieper and Vital [57]. In detail, reads were merged based on RDP’s paired-end assembler and each sequence was assigned a taxonomy using on RDP’s classifier, applying an 80% similarity cut-off value. To obtain detailed insights into the phylotype level, reads were analyzed as described in Schulz et al. [58], where merged reads were aligned within MOTHUR (v1.44.0) [59] using the gotoh algorithm together with the SILVA reference database (v138) and subsequently subjected to the pre.clustering (diff = 2) algorithm. Obtained phylotypes were filtered for an average abundance of ≥0.001% and a sequence length ≥250 bp before analyses and subjected to RDP’s classifier. For detailed taxonomic classifications of the most abundant *Bifidobacteria*-associated phylotypes, respective sequences were aligned with all reference isolates from this taxon available at RDP using Clustal Omega’s webserver (accessed April 2020), and a neighbor joining tree was constructed in MEGA (v7) [60]. Phylotypes closely clustered to respective references allowing for classification at the species level. Follow-up data analyses were conducted in R (v4.1.0), where all samples were resampled to equal the smallest library size of 7775 reads using the *phyloseq* package (v1.36) [61], returning 2644 phylotypes. α-diversities (Richness, Shannon index) were calculated via *phyloseq*, whereas a non-metric multidimensional scaling (NMDS) analysis was conducted on the phylotype level using the package *vegan* (v2.6-2) [62]. Linear regression models (function *lmer* from the package *lme4* (v1.1-3)) were applied on log-transformed count data (log10(count +1)) to calculate the differential abundance of individual taxa during PERT withdrawal (data after reintroduction of PERT were not considered), where animals were included as random effects. Spearman correlation (function *rcorr* from the package *Hmisc* (v4.6)) was used to calculate the association between the two sampling sites (ileal digesta and feces). Only taxa displaying an average relative abundance >0.1% were included in the statistical analyses. FDR-corrected *p*-values < 0.05 (*lfdr* from the package *fdrtool* (v1.2.15)) were considered significant. Plots were created using the package *ggplot2* (v3.3.6).

## 5. Conclusions

Profound community changes occurred rapidly due to enzyme omission affecting α-diversity and relative abundance changes of several taxa of ileal and fecal communities, where in particular *Bifidobacteria* and *Lactobacilli* increased. Changes were reversible after continuing PERT. Dysbiosis events found already in the ileum had accumulating effects in distal parts of the gastrointestinal tract with additional alterations occurring only in the colon.

## Figures and Tables

**Table 1:** Characteristics of the minipigs enrolled in this study.

	PL-Pigs (*n* = 8)		Control Pigs (*n* = 5)	
	Range	Mean ± *SD*	Range	Mean ± *SD*
Age (years)	1–5	3.38 ± 1.30	2–5	3.60 ± 1.34
Body weight (kg)	21.6–29.3	26.4 ± 2.89	24.8–32.6	27.9 ± 4.06
Time after surgery (years)	1–4	2.63 ± 1.06	1–4	2.20 ± 1.30
Chymotrypsin activity in feces (U/g)	0.106–0.424	0.225 ± 0.155	2.4–26.9	10.5 ± 9.70

**Table 2:** Mean abundance of individual bacterial phylotypes that significantly changed during enzyme withdrawal. M_Ildig and M_col refer to the mean relative abundance of all ileal digesta and fecal samples, respectively. Arrows indicate significant changes (pink: ileal digesta, black: feces). Green open circles indicate significant correlations (FDR corrected Spearman’s rho < 0.05) between the two sites. *: for *Bifidobacteria,* the name of the closest matching species based on detailed phylogenetic analyses is given.

ID	Annotation	M_Ildig (%)	M_fec (%)			
2	B. thermophilum *	7.36	7.39	↑	↑	◯
9	B. pseudolongum *	1.95	1.13	↑	↑	◯
10	B. thermophilum *	1.63	1.65	↑	↑	◯
18	B. boum *	1.04	0.52	↑	↑	◯
28	*Clostridium sensu stricto*	0.62	0.43	↓	↓	◯
49	*Holdemanella*	0.43	0.27	↓	↓	◯
99	*Alloprevotella*	0.32	0.01	↓	↓	◯
40	*Clostridium sensu stricto*	0.32	0.40	↓	↓	◯
67	*Selenomonas*	0.30	0.20	↑	↑	◯
70	*Mitsuokella*	0.28	0.22	↑	↑	◯
87	*Clostridium sensu stricto*	0.21	0.12	↓	↓	◯
61	*Prevotellaceae*	0.20	0.37	↑	↑	◯
95	*Romboutsia*	0.16	0.18	↓	↓	◯
132	*Prevotellaceae*	0.09	0.13	↑	↑	◯
108	*Bacteroidales*	0.05	0.22	↓	↓	◯
110	*Solobacterium*	0.05	0.25	↑	↑	◯
109	*Bacteroidales*	0.04	0.24	↓	↓	◯
123	*Parabacteroides*	0.03	0.22	↓	↓	
25	*Prevotella*	1.20	0.10	↑		
37	*Lactobacillus*	0.78	0.04	↓		
42	*Prevotella*	0.72	0.04	↑		
1	*Lactobacillus*	16.41	7.25		↑	◯
5	*Succinivibrio*	1.37	2.90		↓	
4	*Turicibacter*	2.99	2.31		↓	◯
14	*Bacteria*	0.14	2.22		↓	
17	*Bacteria*	0.01	1.82		↓	
16	*Olsenella*	0.46	1.55		↑	◯
20	*Bacteroidetes*	0.06	1.50		↓	
3	*Escherichia/Shigella*	4.22	1.46		↓	
26	*Lachnospiraceae*	0.00	1.35		↓	
19	*Acidaminococcaceae*	0.33	1.06		↑	◯
8	*Enterobacteriaceae*	2.63	0.81		↓	
51	*Bacteria*	0.00	0.72		↓	
48	*Bacteria*	0.07	0.70		↑	◯
39	*Clostridiales*	0.18	0.63		↑	◯
44	*Bacteria*	0.05	0.55		↑	
31	*Lactobacillus*	0.67	0.31		↑	
34	*Lactobacillus*	0.60	0.30		↑	
21	*Lactobacillus*	1.13	0.24		↑	
36	*Lactobacillus*	0.68	0.18		↑	

## Data Availability

Raw sequence data are available at the European Nucleotide Archive [PRJEB51417].
